# Intrathoracic fibrolipoma resected using complete thoracoscopic surgery: a case report

**DOI:** 10.1186/s13019-018-0808-4

**Published:** 2018-11-14

**Authors:** Satoshi Kamata, Itaru Ishida, Yuyo Suzuki, Takehiro Yamada, Hiroshi Yaegashi, Hiroyuki Oura

**Affiliations:** 1 Department of Thoracic Surgery, Iwate Prefectural Central Hospital, Ueda 1-4-1, Morioka, 020-0066 Japan; 2 Department of Pathology, Iwate Prefectural Central Hospital, Ueda 1-4-1, Morioka, Japan

**Keywords:** Intrathoracic surgery, Fibrolipoma, Complete thoracoscopy

## Abstract

**Background:**

Other than adipocytes, lipomas may contain mesodermal components such as varying proportions of fibrous tissues and blood vessels. Fibrolipoma is an uncommon variant of lipoma and comprises a high proportion of fibrous components. An intrathoracic fibrolipoma is extremely rare; to the best of our knowledge, only three such cases have been reported till date.

**Case presentation:**

A 51-year-old female presented with a left intrathoracic mass, which was confirmed to be a lipomatous tumor using computed tomography. A pedunculated tumor originating from the parietal pleura was resected using complete thoracoscopic surgery. Pathological examination indicated a diagnosis of fibrolipoma. Intrathoracic fibrolipomas are extremely rare; this is one of the first reported cases of successfully resecting an intrathoracic fibrolipoma using complete thoracoscopic surgery.

**Conclusions:**

The tumor was asymptomatic and relatively small when detected during a medical checkup. This enabled the successful resection of the tumor via complete thoracoscopic surgery. Although fibrolipomas are histologically benign, careful observation and follow-up are essential owing to the possibility of recurrence.

## Background

Lipomas may contain mesodermal components other than adipocytes, including varying proportions of fibrous tissues and blood vessels. Fibrolipomas are an uncommon variant of lipomas that contain a high proportion of fibrous components. Fibrolipomas are reported to develop during the maturation of lipoblastomatosis; the maturation of adipose and fibrous tissues causes collagen fibers to separate fat cells into lobules, which is a characteristic feature of fibrolipoma [1–3].

Lipomas account for 20% of all benign soft-tissue tumors; however, fibrolipomas are rare and account for only 0.03% of all benign soft-tissue tumors [4]. They can present in any part of the body, including in subcutaneous and muscular soft tissues.

Intrathoracic fibrolipomas are extremely rare. To the best of our knowledge, only three such cases have been reported till date [1, 2, 5]. Herein, we report one of the first cases of an intrathoracic fibrolipoma that was successfully resected during complete thoracoscopic surgery.

## Case presentation

**Fig. 1:**
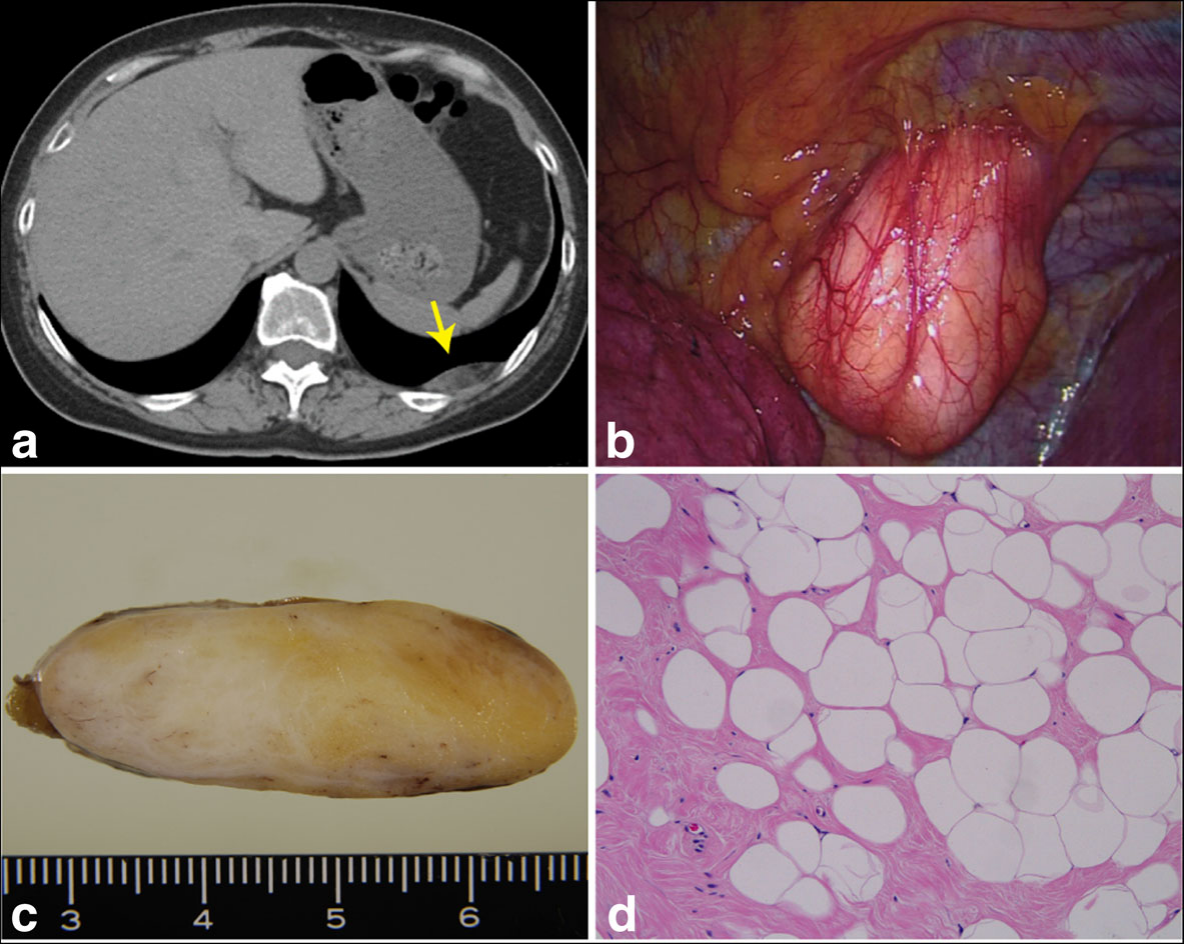
**a** A neoplastic tumor with a smooth margin was observed on the dorsal side in the left lower lung field (arrow). **b** The tumor protruded into the intrathoracic space and was covered by the normal pleura. **c** Macroscopic image of the sliced tumor. **d** Histological findings were consistent with those of fibrolipoma (H&E, × 200)

A 51-year-old female with no subjective symptoms was referred to our hospital with a left intrathoracic mass that was discovered during a medical checkup. Computed tomography (CT) revealed a neoplastic tumor (43 × 48 × 13 mm) with extrapleural signs, located in the dorsal portion of the left thoracic cavity (Fig. 1a). The tumor demonstrated mixed and heterogeneous absorption, which indicated fat and soft tissue. Positron emission tomography (PET) revealed no significant uptake by the tumor. These findings suggested that the mass was a benign lipomatous tumor or lipoma with bleeding originating from the parietal pleura. However, the possibility of a malignant liposarcoma could not be excluded; therefore, we decided to excise the lesion to determine its pathological diagnosis and subsequent treatment.

The patient was intubated using a double-lumen endobronchial tube under general anesthesia and was placed in the right lateral position. After initiating one-lung ventilation, three thoracoscopic ports were placed on the left chest wall, demonstrating a pedunculated mass hanging from the parietal pleura, without attachment to the lung (Fig. 1b). The parietal pleura was incised near the tumor stalk, which allowed easy dissection of the tumor form the chest wall and extirpation via complete thoracoscopic surgery.

The excised specimen was a yellowish-white, soft elastic tumor with a thin fibrous coating (Fig. 1c). Histopathological examination revealed a fibrous component in the tumor as well as dense adipocyte growth (Fig. 1d). Based on these findings, the tumor was diagnosed as a fibrolipoma. The patient experienced no postoperative complications and was discharged on postoperative day 5. After 2 postoperative years, the patient is alive without recurrence.

## Discussion and conclusions

Intrathoracic fibrolipomas are extremely rare, and to the best of our knowledge, only three such cases have been previously reported in the English literature. One of these was associated with the posterior mediastinum, and two were associated with the parietal pleura [1, 2, 5].

Lipomas and their variants exhibit attenuation of fat of approximately − 100 HU on CT images. Moreover, magnetic resonance imaging (MRI), particularly with fat saturation, is helpful for assessing the lipomatous nature of the tumor. In addition, MRI aids in distinguishing between lipomas and well-differentiated liposarcomas based on margins, signal homogeneity, and septa or nodules [6]. PET imaging may also be an objective and useful modality for preoperatively evaluating tumors involving adipose tissue. In the present case, the tumor was considered to be a benign lipomatous tumor, rather than a malignant liposarcoma, following confirmation using CT and PET images. However, completely excluding liposarcoma can be difficult. For preoperative diagnosis, additional MRIs may provide information regarding tumor descriptions; moreover, histological evaluation using core needle biopsy could be performed. Conversely, the differentiation of various histological types of lipomatous tumors is not easy, particularly when limited tissue is available for assessment. Therefore, in the present case, we decided to completely resect the lesion, without obtaining an MRI scan or performing core needle biopsy.

In the present case, intraoperative, frozen-section analysis revealed that the tumor was rich in fibrous components and demonstrated no malignancy. Generally, benign lipomas and liposarcomas can be distinguished depending on the presence of hyperchromatic nuclei; however, the identification of atypical stromal cells is not always easy. Thus, establishing a differential diagnosis using only intraoperative frozen-section analysis is difficult for pathologists. Because the differentiation of lipomas and liposarcomas is essential for proper patient management, it is important to conduct a detailed examination of the whole resected specimen.

The lipomatous tumors exhibit varied clinical course. The first-line treatment for these tumors involves surgical resection. The prognosis is favorable for benign lipomas and well-differentiated liposarcomas after complete resection. However, high-grade tumors such as pleomorphic liposarcomas may develop distant metastases.

Fibrolipomas present clinical characteristics similar to those of lipomas. Intrathoracic fibrolipomas are usually asymptomatic; however, the development of tumor causes the compression of surrounding organs, resulting in a shortness of breath and dysphagia [3]. In the three previously reported cases of intrathoracic fibrolipomas, the tumors were relatively large (> 8 cm) but asymptomatic. Fibrolipomas are commonly capsulated, and those originating from the parietal pleura are usually pedunculated, which facilitates an easy separation of fibrolipomas from surrounding organs. In previously reported cases, standard thoracotomies were necessary to excise the tumors owing to their large sizes.

In the present case, the tumor was asymptomatic and was relatively small upon when first detected. Therefore, successfully resecting the tumor using complete thoracoscopic surgery was possible. Although fibrolipomas are histologically benign, careful observation and follow-up are essential owing to the possibility of recurrence [7].

## Abbreviations

CT: Computed tomography
MRI: Magnetic resonance imaging
PET: Positron emission tomography
